# Effects of Slip Length and Inertia on the Permeability of Fracture with Slippery Boundary Condition

**DOI:** 10.3390/ijerph17113817

**Published:** 2020-05-28

**Authors:** Benhua Liu, Hao Zhan, Yiran Liu, Huan Qi, Linxian Huang, Zhengrun Wei, Zhizheng Liu

**Affiliations:** 1 School of Water Conservancy and Environment, University of Jinan, Jinan 250022, China; zhanh_edu@163.com; 2 Engineering Technology Institute for Groundwater Numerical Simulation and Contamination Control, Jinan 250022, China; 3 School of Water Resources and Environment, China University of Geosciences (Beijing), Beijing 100083, China; 2005190030@cugb.edu.cn; 4 801 Institute of Hydrogeology and Engineering Geology, Shandong Provincial Bureau of Geology & Mineral Resources, Jinan 250014, China; qihuan6688@outlook.com; 5 Shandong Institute of Geological Survey, Jinan 250013, China; w5029@163.com (Z.W.); liuzhizheng1985@126.com (Z.L.)

**Keywords:** slip length, inertial force, fracture, nonlinear flow, permeability

## Abstract

Although the slippery boundary condition (BC) has been validated to enhance fracture permeability (*k*), the coupling effects of heterogeneous slippery BC and inertia on *k* remain less understood. We used computational fluid dynamics to investigate the competing roles of slippery BC and inertial forces in controlling *k* evolution with increasing pressure gradient by designing six cases with different slip length scenarios for a two-dimensional natural fracture. Our results suggest that pronounced inertial effects were directly related to and demonstrated by the growth of recirculation zone (RZ); this caused flow regimes transitioning from Darcy to non-Darcy and significantly reduced *k*, with an identical tailing slope for six cases, regardless of the variability in slip lengths. Moreover, the slippery BC dominantly determine the magnitude of *k* with orders depending on the slip length. Lastly, our study reveals that the specific *k* evolution path for the case with a varying slip length was significantly different from other cases with a homogeneous one, thus encouraging more efforts in determining the slip length for natural fractures via experiments.

## 1. Introduction

Fracture flow is critical for the success of many environmental and engineering applications, including the exploration of oil and gas and geothermal energy [1,2], groundwater contamination remediation [3,4,5,6,7], and geological carbon sequestration [8,9,10,11]. Essentially, the fluid-borne contaminants travelling along with subsurface fluid flow are normally hard to predict, where non-Fickian transport phenomenon is pervasive [3,5]. In such a circumstance, a reliable characterization of subsurface flow fields is the key to accurately capture the transport process [12]. For fractures, although the traditional fracture flow theory, namely Cubic Law [13], or the recently modified Local Cubic Law considering fracture heterogeneity [14,15], have been widely used to predict fracture flow, how inertial effects come to play, and change fracture permeability remain less constraint.

Inertial effects cannot be ignored with increasing pressure gradient, driving flow regime transitioning from Darcy to non-Darcy [16,17,18]. Consequently, permeability declines with increasing inertial forces, which makes it difficult to accurately predict (non-Darcy) fracture flow. To overcome the challenge in describing non-Darcy flow in fractured and porous media, a recent study proposed a universal relationship between inertial and viscous permeability based on thousands of laboratory experimental results [19]. With the established universal relationship, one can easily predict fracture nonlinear flow, but with several orders of magnitude of uncertainties that need to be refined.

We proposed that one of possible reasons resulting in large uncertainty is the slippery boundary condition (BC). Slippery BC could be caused either by the chemical alteration or by the coverage of wetting film on the solid surfaces. For instance, many studies reported that the rock surface can be switched from hydrophilic to hydrophobic after its exposure to a non-wetting fluid [20]. The slippery BC, in fact, has been tested to enhance fracture flow (i.e., permeability) via both laboratory and numerical experiments [20,21]. Note that the enhanced fracture flow might not increase the longitudinal dispersion coefficient within the simplified parallel plate model [21,22] for the first time investigated the competition between the inertial force and slippery BC effects in changing fracture permeability, where inertial force on the one hand reduces permeability, while slippery BC, on the other hand, enhances permeability. By employing a homogeneous slip length along fracture boundary, they found out that slippery effects dominated over inertial effects when the pressure gradient was small, and the inertial effects gradually caught up with that caused by the slippery BC with a further increase in pressure gradient [21]. Nonetheless, how heterogeneous slippery BC (i.e., slip length varies along fracture length) alters permeability with increasing pressure gradient remain less understood.

In this study, we aim to address following questions: (1) how does the heterogeneous slippery BC alter the flow regimes transitioning from Darcy to non-Darcy; (2) is there an equivalent homogeneous slip BC that can be used in lien of heterogeneous slippery BC in terms of permeability alteration; (3) how are the recirculation zones (RZs) that are caused by inertial effects developing and growing that could be further complicated by heterogeneous slippery BC. To this end, we resorted to computational fluid dynamics to quantify RZ growth and permeability evolution with increasing pressure gradient.

## 2. Methodology

### 2.1. Fracture Data and Computational Fluid Dynamics

**Figure 1:**
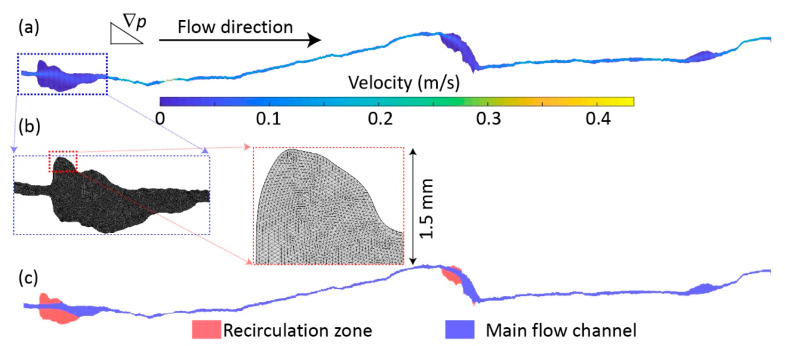
(**a**) The flow field driven by the pressure gradient (∇p) from left (upstream) to right (downstream) with velocity magnitude illustrated by the filled color. (**b**) A typical segment of fracture showing the mesh scheme used for computational fluid dynamics. (**c**) The recirculation zone (red zone) induced by significant inertial effects and main flow channel (blue zone) are delineated by the code proposed by [23].

For the purpose of gaining fundamental knowledge without losing generality, we used a two-dimensional (2D) fracture (Figure 1a), extracted from a three-dimensional fracture that was obtained from the Digital Rocks Portal (https://www.digitalrocksportal.org/), where the welded Santana tuff sample has been used to investigate the fluid flow and solute transport processes [5,14,21]. The fracture was scanned by X-ray tomography with a resolution of about 0.25 mm done at The University of Texas at Austin, with a length of about 15 cm and mean aperture of about 6.2 × 10**^−^**^4^ m. More fracture information can be found at reference [14].

Fluid flow through fractures is essentially governed by the Navier–Stokes equations (NSE), along with a continuity equation:

(1) $$\rho \left(u\cdot \nabla \right)u=-\nabla p+\mu \nabla^{2}u$$

(2) ∇·u=0

where *ρ* (1000 kg/m^3^) is the fluid density, **u** is the velocity vector, *μ* (0.001 Pa⋅s) is fluid dynamic viscosity, and *p* is total pressure. The NSE were directly solved, including an inertial term on the left-hand side to precisely capture the growth of recirculation zones (RZs) with increasing pressure gradient.

In this study, the NSE were solved by computational fluid dynamics via the commercial software COMSOL Multiphysics that is built upon the finite element method. To solve the NSE, a fracture domain was discretized with a minimum mesh size of 30 μm around the fracture walls and mesh size of 60 μm towards to the middle of domain (Figure 1b), resulting more accurate numerical solutions (e.g., velocity field in Figure 1a) around the boundaries. Moreover, we applied a specified pressure boundary condition at both upstream and downstream (i.e., Dirichlet boundary condition), driving fluid flowing from left to right (Figure 1a). We sequentially increased the pressure gradient (∇p was up to 6000 Pa/m with an uneven interval of about 50 Pa/m) until RZs were fully developed to be able to capture considerable inertial effects (Figure 1c).

In terms of slippery boundary condition (BC), we followed previous study [21] by specifying a slip length (*L**_s_***) along the fracture walls. Different from previous study, we considered six cases with different *L**_s_***: (1) Case 1: No slip case with *L**_s_*** equal to 0 m; (2) Case 2: *L**_s_*** was homogeneous and set to be the minimum value in the case 3; (3) Case 3: *L**_s_*** varies along the fracture length that depends on the fracture aperture field following the equation proposed by [21]; (4) Case 4: *L**_s_*** was homogeneous and set to be the arithmetic mean value in the case 3; (5) Case 5: *L**_s_*** was homogeneous and set to be the median value in the case 3; and, (6) Case 6: *L**_s_*** was homogeneous and set to be the maximum value in the case 3. The six cases enable us to further distinguish the competing effects that are caused by the inertial and slippery BC on permeability.

### 2.2. Automatic Recirculation Zone Quantification

Inertial effects can lead to permeability reduction, which is directly related to development and growth of RZs inside the pores and fractures [16,21]. We resorted to the open source of MATLAB code to be able to capture and quantify the growth of RZs (https://figshare.com/articles/Automatic_detection_of_2D_recirculation_zone_volume_/5811399). More details about the delineation of RZs can be found in previous study [23].

### 2.3. Fracture Permeability Analysis

Upon solving the NSE, we computed fluxes (*Q*) for above-mentioned six cases by integrating numerically derived velocity at the downstream of fracture. With knowing *Q*, the permeability (*k*) can be estimated by:

(3) $$k=\frac{{\left(\frac{12\mu Q}{\nabla p}\right)}^{2/3}}{12}\;$$

where ∇p is pressure gradient; and, the Reynolds number in this study is defined as:

(4) $$Re=\frac{\rho \langle v\rangle b}{\mu }=\frac{\rho Q}{\mu }$$

where ⟨v⟩ is the mean velocity within the fracture domain and *b* is mean fracture aperture.

## 3. Results and Discussion

### 3.1. Fracture Nonlinear Flow Behavior

**Figure 2:**
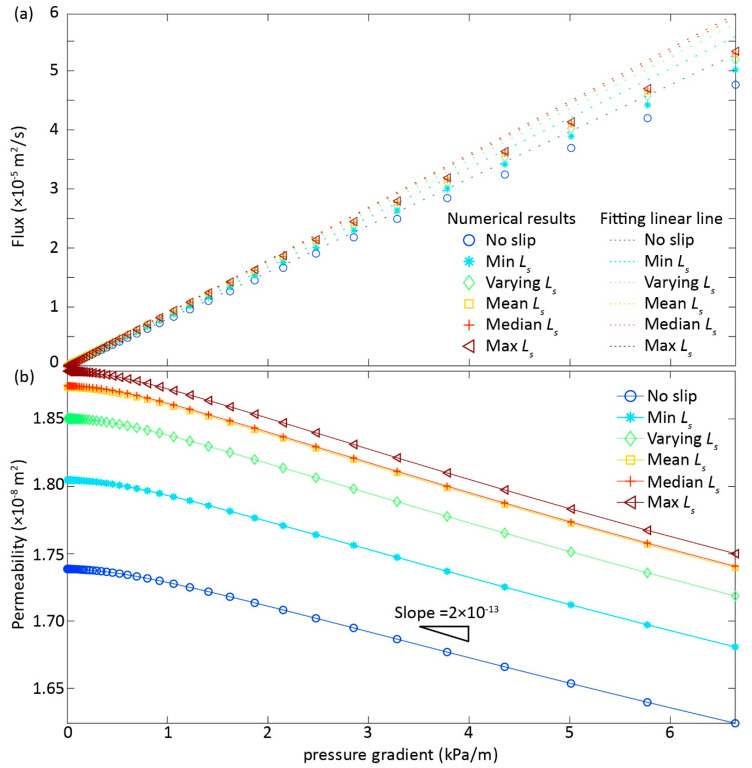
The relationships of flux (**a**) and permeability (**b**) with pressure gradient for six cases with a variety of slip lengths *L_s_*: case 1—no slip length with *L_s_* equal to 0; case 2—minimum *L_s_* assigned in the case 3; case 3—varying *L_s_* depending on aperture based on [20,21]; case 4—mean *L_s_* assigned in the case 3; case 5—median *L_s_* assigned in the case 3; and, case 6—maximum *L_s_* assigned in the case 3. In (**a**), the dashed-lines represent the linear fitting curves to the numerical-derived results (symbols). In (**b**), the slope value was estimated by dividing the fracture permeability by pressure gradient.

The flow transitioning from Darcy (linear) to non-Darcy (nonlinear) regimes occurs when the inertial effects are non-negligible [17,19]. Consistent with previous studies, our computational results under increasing ∇*p* clearly show that ∇p *− Q* curves deviate from the linear fitting line (Figure 2a), where *Q* is less than the linear proportionality as Darcy’ s law prescribes due to additional inertial energy dissipation [19]. Noticeably, the largest degree of flow nonlinearity deviating from linear Darcy regime is the no-slip case, as expected, followed by the case 2 with a minimum *L_s_* along fracture length due to the enhancement of flow field by slippery boundary condition (BC), and further followed by the cases 3–6 with spatially varying, maximum, mean, and median *L_s_*, respectively (Figure 2a).

An alternative way to diagnose the flow regime transition is to check the *k* evolution path with an increase in ∇p. *k* remains constant if Darcy’s law holds, and decreases due to additional inertial energy dissipation with increasing ∇p, indicating flow behavior transitioning from Darcy to non-Darcy regimes [16]. We estimated *k* based on the numerically derived *Q*, which directly suggests the emergence of nonlinear flow regime (Figure 2b). Congruent with results inducing from ∇p *− Q* curves, the case 1 with no-slip BC has the least *k*, followed by the minimum *Ls* case 2, and by the cases 3−5, with the maximum *k* for the case 6. Again, the reduction of *k* with increasing ∇p for different cases can be explained by the extra inertial forces with the magnitude of *k* reduction, depending on *L_s_*. Interestingly, the slopes of *k* − ∇p were almost identical with a further increase of ∇p (∇p > 1000 Pa/m), regardless of variability in *L_s_* specification, suggesting that the inertial effects were significant and might dominate over slippery BC effects in determining the *k* evolution paths.

### 3.2. Inertial Effects on Reducing Permeability

**Figure 3:**
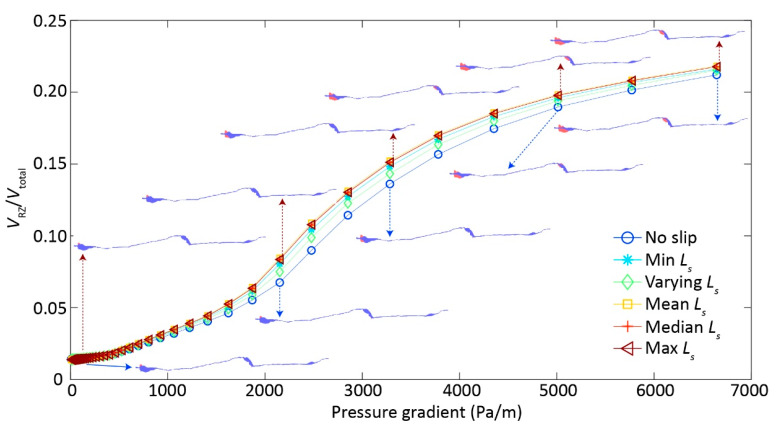
The recirculation zone volume ratio (*V*_RZ_/*V*_total_) increases with pressure gradient for six cases with a variety of slip lengths *L_s_*: case 1—no slip length with *L_s_* equal to 0; case 2—minimum *L_s_* assigned in the case 3; case 3—varying *L_s_* depending on aperture based on [20,21]; case 4—mean *L_s_* assigned in the case 3; case 5—median *L_s_* assigned in the case 3; and, case 6—maximum *L_s_* assigned in the case 3, where *V*_RZ_ is circulation volume, *V*_total_ is the total fracture volume. Insets illustrates the spatial distribution of recirculation zones with increasing pressure gradient for the no-slip case 1 (blue arrows) and case 6 (brown arrows).

The inertial effects were more pronounced with increasing ∇p and, thus, led to the overall *k* reduction behavior (Figure 2b). We resorted to the estimation of recirculation zone (RZ) volume to be able to quantify inertial effects (Figure 1c), since RZ is directly related to inertial effects [16,21]. Expectedly, RZ volume increased with ∇p where inertial effects gradually increased (Figure 3), leading to *k* reduction with ∇p (Figure 2b). Note that the case 1 with no-slip BC eventually had the least RZ volume when ∇p approached to 6500 Pa/m; the RZ volume increased from the case 2 to case 3, with the largest and almost identical RZ volume for the cases 4−6 (Figure 3).

**Figure 4:**
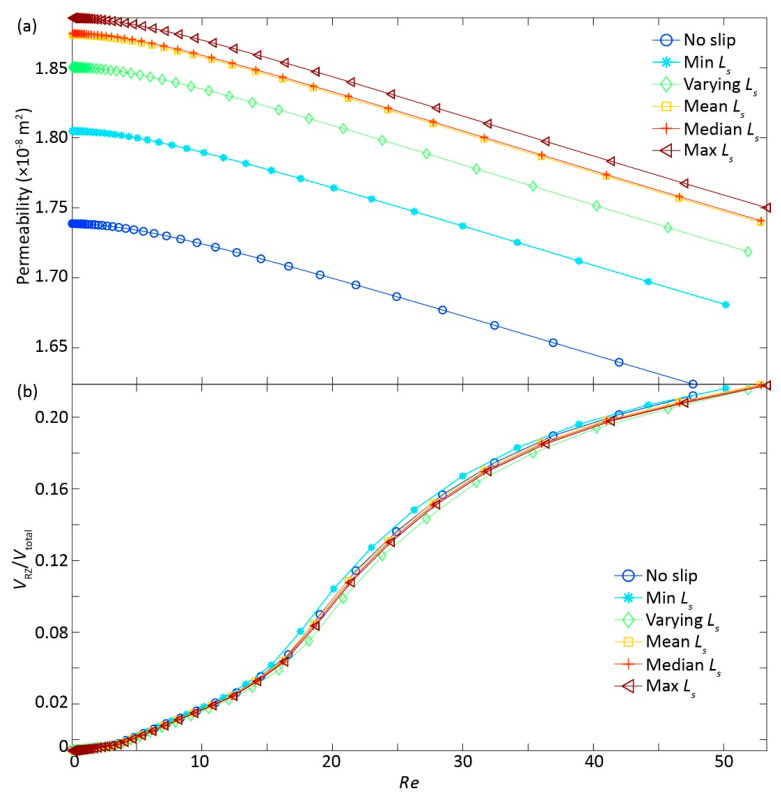
The relationships of permeability (**a**) and recirculation zone volume ratio (*V*_RZ_/*V*_total_) (**b**) with Reynolds number (*Re*) for six cases with a variety of slip lengths *L_s_*: case 1—no slip length with *L_s_* equal to 0; case 2—minimum *L_s_* assigned in the case 3; case 3—varying *L_s_* depending on aperture based on [20,21]; case 4—mean *L_s_* assigned in the case 3; case 5—median *L_s_* assigned in the case 3; and, case 6—maximum *L_s_* assigned in the case 3, where *V*_RZ_ is circulation volume, *V*_total_ is the total fracture volume.

When considering the intricate competition between inertial and slippery BC effects [21], we replotted *k* and RZ volume against Reynolds number (*Re*) (Figure 4a,b). Alike *k* ~ ∇p relationship (Figure 2b), *k* ~ *Re* curves followed the linear tailing behavior with differences depending on *L_s_* specification. However, unlike the ∇p ~ RZ volume relationship (Figure 3), there was little difference in the *Re* ~ RZ volume curves between the cases with varying *L_s_* (Figure 4b), suggesting the RZ volume was fundamentally controlled by the fluid dynamics, as quantified by the dimensionless number *Re*, where both inertial and slippery effects come to play a role in determining RZ volume. This is congruent with previous study [21], where the roles of inertia and slippery BC are competing with each other in determining *k*. Moreover, the competing roles might also lead to the variability in the threshold of *Re* for identifying the transition from Darcy to non-Darcy flow regimes [24], which warrants future study.

### 3.3. Slippery Effects on Enhancing Permeability

The slippery BC can undoubtedly accelerate the flow velocity by lessening flow resistance along the fracture walls [20] and, thus, enhanced fracture permeability with a given ∇p (Figure 2 and Figure 3). Consequently, the degree of flow nonlinearity with increasing ∇p was dampened by an increase of *L_s_* (Figure 2a), resulting in the highest *k* for the case 6, followed by the case 5 till to the case 1 (Figure 2b) with a given ∇p. This is somehow expected, because case 6 with the largest *L_s_* was supposed to have the largest *k* with a given ∇p, and vice versa for the case 1 with no-slip BC (Figure 2b and Figure 4a).

While expected for the *k* enhancement with slippery effects, the RZ growth behavior with increasing ∇p cannot not fully explained by the order of *L_s_* values (Figure 3); that is, the case 2 with the minimum *L_s_* was expected to have less RZ volume than the case 3 with heterogeneous (varying) *L_s_*, which was not the case here. The reversed order of RZ volume in the cases 2−3 can be reasonably explained by the complex interplay between inertial and slippery effects, because: (1) the RZ favorably grows in the small aperture areas right behind the large area zone [25], where *L_s_* was small corresponding to the small aperture, thus possibly leading to an equivalently-effective *L_s_* in enhancing RZ growth for the cases 2−3; (2) the slippery effects can be dampened or overshadowed by the inertial effects [21], thus a large *L_s_* can possibly have a small RZ volume. Although RZ volume demonstrated a complex relationship with ∇p, the rest of fractures have the expected order of RZ volumes following the magnitude of *L_s_*, because inertial effects were still weak when compared to the slippery effects, suggesting that the overall *k* magnitude was controlled by the *L_s_* (Figure 2, Figure 3, Figure 4).

**Figure 5:**
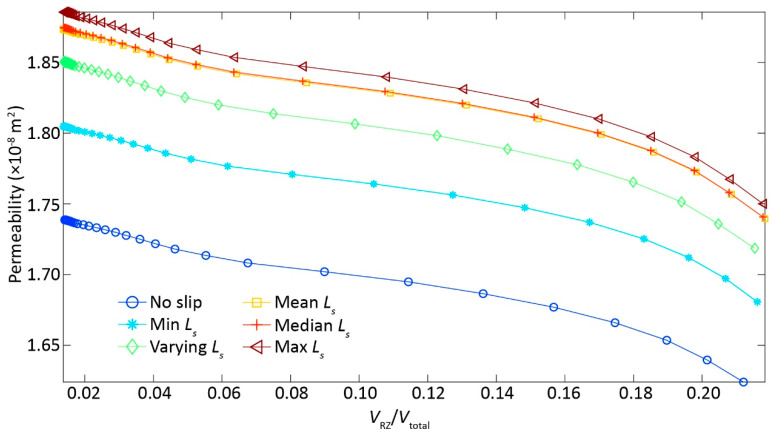
The relationship of permeability with recirculation zone volume ratio (*V*_RZ_**/***V*_total_) for six cases with a variety of slip lengths *L_s_*: case 1—no slip length with *L_s_* equal to 0; case 2—minimum *L_s_* assigned in the case 3; case 3—varying *L_s_* depending on aperture based on [20,21]; case 4—mean *L_s_* assigned in the case 3; case 5—median *L_s_* assigned in the case 3; and, case 6—maximum *L_s_* assigned in the case 3, where *V*_RZ_ is circulation volume, *V*_total_ is the total fracture volume.

We plotted *k* against RZ volume to further demonstrate this argument and clarify this complexity in the *k* evolution (Figure 5). Clearly, *k* decreased with RZ volume (i.e., inertial effects) with different trajectories depending on the *L_s_*; that is, the magnitude of *k* with RZ volume followed the order of *L_s_*, with the largest *k* for case 6, followed by the cases from 5 to 1. This sequence was not interfered by the reversed order (cases 2 and 3) of RZ volume with increasing *L_s_* (Figure 3), suggesting the dominant role of *L_s_* in controlling *k*.

Finally, our results reveal that the realistic case (case 3) with the varying *L_s_* along fracture walls cannot be replaced and investigated using a homogeneous with either minimum, mean, median, or maximum values of *L_s_*, thus encouraging more experimental insights into the determination of *L_s_* for natural fractures.

## 4. Conclusions

We investigate the roles played by the slippery effects and inertial effects with increasing pressure gradient in controlling fracture permeability (*k*) by designing six numerical experiments with different slip lengths for a two-dimensional fracture. We found that the inertial effects can significantly reduce *k* with emergent growing recirculation zones (RZs) inside the fracture. Unlike inertial effects, slippery effects can enhance fluid flow and *k*, thus competition between inertial and slippery effects was demonstrated by the growth of RZs. Moreover, slippery effects overall controls the magnitude of *k* over the course of *k* reduction with increasing pressure gradient, where the more realistic varying slip length case cannot be represented by using any homogeneous one. Therefore, urgent effort is required to measure the spatial distribution of slip length via laboratory experiments to accurately predict fluid flow through fractures considering slippery effects.

## References

[1] Christensen M., Tanino Y. (2017). Enhanced permeability due to apparent oil/brine slippage in limestone and its dependence on wettability. Geophys. Res. Lett..

[2] Zheng L., Wang L. (2019). Scale-dependent Poiseuille flow alternatively explains enhanced dispersion in geothermal environments. Hydrol. Process..

[3] Zheng L., Wang L., James S.C. (2019). When can the local advection–dispersion equation simulate non-Fickian transport through rough fractures?. Stoch. Environ. Res. Ris. Assess..

[4] Yang Z., Niemi A., Fagerlund F., Illangasekare T., Detwiler R.L. (2013). Dissolution of dense non-aqueous phase liquids in vertical fractures: Effect of finger residuals and dead-end pools. J. Contam. Hydrol..

[5] Wang L., Cardenas M.B. (2014). Non-Fickian transport through two-dimensional rough fractures: Assessment and prediction. Water Resour. Res..

[6] Neuman S.P. (2005). Trends, prospects and challenges in quantifying flow and transport through fractured rocks. Hydrogeol. J..

[7] Nowamooz A., Radilla G., Fourar M. (2009). Non-Darcian two-phase flow in a transparent replica of a rough-walled rock fracture. Water Resour. Res..

[8] Berkowitz B. (2002). Characterizing flow and transport in fractured geological media: A review. Adv. Water Resour..

[9] Wang L., Cardenas M.B. (2017). Linear permeability evolution of expanding conduits due to feedback between flow and fast phase change. Geophys. Res. Lett..

[10] Elkhoury J.E., Ameli P., Detwiler R.L. (2013). Dissolution and deformation in fractured carbonates caused by flow of CO_2_-rich brine under reservoir conditions. Int. J. Greenh. Gas. Cont..

[11] Wang L., Cardenas M.B. (2018). Connecting Pressure-Saturation and Relative Permeability Models to Fracture Properties: The Case of Capillary-Dominated Flow of Supercritical CO_2_ and Brine. Water Resour. Res..

[12] Huang L., Wang L., Shao J., Liu X., Hao Q., Xing L., Zheng L., Xiao Y. (2018). Parallel Processing Transport Model MT3DMS by Using OpenMP. Int. J. Environ. Res. Public Health.

[13] Witherspoon P.A., Wang J.S.Y., Iwai K., Gale J.E. (1980). Validity of Cubic Law for fluid flow in a deformable rock fracture. Water Resour. Res..

[14] Wang L., Cardenas M.B., Slottke D.T., Ketcham R.A., Sharp J.M. (2015). Modification of the Local Cubic Law of fracture flow for weak inertia, tortuosity, and roughness. Water Resour. Res..

[15] Brush D.J., Thomson N.R. (2003). Fluid flow in synthetic rough-walled fractures: Navier-Stokes, Stokes, and local cubic law simulations. Water Resour. Res..

[16] Chaudhary K., Cardenas M.B., Deng W., Bennett P.C. (2011). The role of eddies inside pores in the transition from Darcy to Forchheimer flows. Geophys. Res. Lett..

[17] Qian J., Chen Z., Zhan H., Guan H. (2011). Experimental study of the effect of roughness and Reynolds number on fluid flow in rough-walled single fractures: A check of local cubic law. Hydrol. Process..

[18] Qian J., Zhan H., Chen Z., Ye H. (2011). Experimental study of solute transport under non-Darcian flow in a single fracture. J. Hydrol..

[19] Zhou J.-Q., Chen Y.F., Wang L.C., Cardenas M.B. (2019). Universal Relationship between Viscous and Inertial Permeability of Geologic Porous Media. Geophys. Res. Lett..

[20] Lee H.-B., Yeo I.W., Lee K.-K. (2007). Water flow and slip on NAPL-wetted surfaces of a parallel-walled fracture. Geophys. Res. Lett..

[21] Zhou J.-Q., Wang L., Li C., Tang H., Wang L. (2020). Effect of fluid slippage on eddy growth and non-Darcian flow in rock fractures. J. Hydrol..

[22] Zheng L., Wang L., Wang T., Singh K., Wang Z.-L., Chen X. (2020). Can homogeneous slip boundary condition affect effective dispersion in single fractures with Poiseuille flow?. J. Hydrol..

[23] Zhou J.-Q., Wang L., Chen Y.-F., Cardenas M.B. (2019). Mass Transfer between Recirculation and Main Flow Zones: Is Physically Based Parameterization Possible?. Water Resour. Res..

[24] Zhou J.Q., Wang M., Wang L.C., Chen Y.F., Zhou C.B. (2018). Emergence of nonlinear laminar flow in fractures during shear. Rock Mech. Rock Eng..

[25] Cardenas M.B., Slottke D.T., Ketcham R.A., Sharp J.M. (2007). Navier-Stokes flow and transport simulations using real fractures shows heavy tailing due to eddies. Geophys. Res. Lett..

